# Exosome mediated biological functions within skeletal microenvironment

**DOI:** 10.3389/fbioe.2022.953916

**Published:** 2022-07-22

**Authors:** Zhikun Wang, Zhonghan Zhao, Bo Gao, Lingli Zhang

**Affiliations:** ^1^ School of Kinesiology, Shanghai University of Sport, Shanghai, China; ^2^ Institute of Orthopedic Surgery, Xijing Hospital, Fourth Military Medical University, Xi’an, China; ^3^ College of Athletic Performance, Shanghai University of Sport, Shanghai, China

**Keywords:** skeletal related exosomes, bone marrow mesenchymal stem cell, osteoblast, osteoclast, skeletal microenvironment, osteocyte

## Abstract

Exosomes are membranous lipid vesicles fused with intracellular multicellular bodies that are released into the extracellular environment. They contain bioactive substances, including proteins, RNAs, lipids, and cytokine receptors. Exosomes in the skeletal microenvironment are derived from a variety of cells such as bone marrow mesenchymal stem cells (BMSCs), osteoblasts, osteoclasts, and osteocytes. Their biological function is key in paracrine or endocrine signaling. Exosomes play a role in bone remodeling by regulating cell proliferation and differentiation. Genetic engineering technology combined with exosome-based drug delivery can therapy bone metabolic diseases. In this review, we summarized the pathways of exosomes derived from different skeletal cells (i.e., BMSCs, osteoblasts, osteocytes, and osteoclasts) regulate the skeletal microenvironment through proteins, mRNAs, and non-coding RNAs. By exploring the role of exosomes in the skeletal microenvironment, we provide a theoretical basis for the clinical treatment of bone-related metabolic diseases, which may lay the foundation to improve bone tumor microenvironments, alleviate drug resistance in patients.

## 1 Introduction

Although seemingly simple and inert, bones are surprisingly complex and busy tissue. It has been found that abnormal proliferation and differentiation of bone marrow mesenchymal stem cells (BMSCs), osteoblasts, osteocytes and osteoclasts can lead to a variety of bone diseases such as osteoporosis, osteoarthritis, spinal tuberculosis, bone tumors, and osteosclerosis (Zhou et al., 2019). Osteoporosis is a common systemic metabolic bone disease in the elderly, mainly characterized by decreased bone mass, increased bone fragility and bone microstructure destruction (Johnston and Dagar, 2020). Osteoarthritis is the most common chronic degenerative joint disease and a leading cause of pain and disability. The incidence of the disease grows as people age, and its prevalence is steadily increasing and is projected to become the largest cause of disability by 2030 (Cross et al., 2014). The main pathological feature of spinal tuberculosis is progressive bone destruction. The prevalence rate of spinal tuberculosis is the number one in all bone and joint tuberculosis. Bone is one of the most common sites of systemic metastases, accounting for 15–20% of metastatic tumors. Spinal metastases occur in approximately 36% of patients who die of malignancy, while primary bone and soft tissue tumors account for 2–3% of systemic tumors. Malignant bone tumors can either originate from other tissues or organs in the body and metastasize to the bone or directly invade the bone through blood circulation and lymphatic system. Malignant bone tumors develop rapidly with high mortality (Lambert et al., 2017; Chen et al., 2021). Rickets is a metabolic bone disease in children characterized by the failure to mineralize growth plates and an osteoid matrix (Munns et al., 2016). Osteosclerosis is complications of myeloproliferative tumors. These diseases lead to excessive growth of bone trabeculae and collagen fibers that replace hematopoietic cells, resulting in abnormal bone marrow function (Mankar et al., 2020). Bone disorders are caused by congenital or acquired factors that destroy and interfere with normal bone metabolism and biochemical status, resulting in bone biochemical metabolism disorders. To meet the clinical needs of orthopedic diseases, exosomes become a new therapeutic strategy.

Current treatments for bone-related metabolic diseases are limited by poor therapeutic effects, adverse events, failure to improve bone absorption, abnormal bone growth and mineral deposition, and difficulty in reversing the bone disease (Tsai et al., 2019). Stem cell transplantation for osteoporosis treatment has shown preliminary feasibility and efficacy results in mice models; however, there were issues with immune rejection, increased risk of cell malignancy, and stem cell homing limit the adoption of such treatment (Aghebati-Maleki et al., 2019). Therefore, it is necessary to find a targeted treatment for bone metabolic diseases that can minimize the potential harm caused by long-term drug exposure.

Exosomes are nanoscale vesicle particles secreted by cells, which have biological activities similar to those of the source cells and play an important role in intercellular communication (Tkach and Thery, 2016). Studies have found that exosomes derived from skeletal cells participate in skeletal cell proliferation and differentiation (Chen et al., 2017; Song et al., 2019; Xie et al., 2020). Exosomes have high stability, no immunogenicity and strong targeting ability, which make up for the deficiency of traditional drugs and stem cell therapy. A variety of inclusions carried by exosomes can directly act on these skeletal cells. Therefore, the study on the involvement of exosomes derived from various skeletal cells in the regulation of skeletal cell proliferation and differentiation can provide a theoretical basis for the study of bone diseases, and has certain clinical significance for the diagnosis and treatment of bone diseases.

## 2 Characteristics of skeleton-derived exosomes

Exosome is a vesicle with a double-layer lipid membrane structure. It is spherical or cup-shaped, with a diameter of about 40–100 nm and a density of about 1.13–1.21 g/ml (Tkach and Thery, 2016). Exosome contains nucleic acids, which regulate physiological and pathological processes (Zhang et al., 2015; Tkach and Thery, 2016). Non-specific expression proteins found in the exosomes can be used as the protein marker to identify exosomes, including heat shock protein, tumor susceptibility gene101 (TSG101), four-molecule cross-linked transmembrane protein superfamily (CD9, CD63, CD81, CD82), and ALG-2-interacting protein (Alix) (Tkach and Thery, 2016). Moreover, some specific expression proteins are cell-derived and related to cell signal transduction (Zhong et al., 2021).

Exosome play a key role in paracrine or endocrine signaling. The proteins, lipids and nucleic acids contained inside the exosomes have specific biological functions. Exosome transmits information acts on target cells by either targeting cells *via* their receptors (Mathivanan et al., 2010) or entering the target cell *via* endocytosis. The exosomes carry proteins, lipids and nucleic acids into the target cell and activate relevant signaling pathways (Mathivanan et al., 2010).

Exosome are involved in cell proliferation and differentiation, therefore play an important role in bone tissue engineering. The composition of bone-derived exosomes differs from exosomes originated from other sources. During bone reconstruction, bone-derived exosomes may release proteins involved in bone formation, such as alkaline protease (ALP), bone morphogenetic protein (BMP), eukaryotic translation initiation factor2 (elF2), osteopontin (OPN), bone sialoprotein (BSP), and osteocalcin (OCN). Bone-derived exosomes also contain osteoclast differentiation-related proteins such as the receptor activator of nuclear factor κB–ligand (RANKL) and RANK (Huynh et al., 2016). In addition, microRNAs (miRNAs)-related to bone remodeling, such as miR-24, let-7, miR-143-3p, miR-10b-5p, miR-199b, miR-218, and miR-214-3p were also found in bone-derived exosomes (Huynh et al., 2016; Li et al., 2016). These miRNAs have a crucial role in bone formation and resorption. Bone-associated exosomes can selectively transport specific information, depending on the proteins or factors within the exosomes. Pathological conditions, such as inflammation, hypoxia, and pH changes (Deng et al., 2017), can affect exosome release.

## 3 BMSC-Exos-mediated communication within the skeletal micro-environment

BMSCs are stem cells derived from the mesoderm that play an important role in bone regeneration and repair. They also have paracrinal function (Eirin et al., 2017). We previously introduced the characteristics and functions of exosomes derived from BMSC (BMSC-Exos) in detail (Wang Z. et al., 2021). BMSC-Exos enter target cells through endocytosis and plays a bioactive role, showing tissue repair ability similar to BMSCs (van der Pol et al., 2012). Moreover, due to its low immunogenicity and low cellular activity, it is safer than transplantation of stem cells (Liu et al., 2015).

### 3.1 Effects of BMSC-Exos on osteoblasts

Osteoblasts are functional cells that secrete bioactive substances regulating bone formation and remodeling. To detect the effect of BMSC-Exos on osteoblast proliferation, the proliferation curves of osteoblasts stimulated by different concentrations of BMSC-Exos were plotted. Osteoblast proliferation rates were significantly increased after BMSC-Exos were added and were proportional to the concentration of BMSC-Exos (Zhang et al., 2021). These results suggest that BMSC-Exos effectively promote osteoblast proliferation in a dose- and time-dependent manner.

Early differentiation of BMSC into osteoblasts, and mature osteoblasts undergo further mineralization, are both important evaluation indicators of osteogenic differentiation. ALP activity is a marker of early differentiation in osteoblasts (Osugi et al., 2012). OCN is essential in regulating bone conversion and mineralization, and often used as a marker of bone formation in late stage (Tsao et al., 2017). Both runt-related transcription factor 2 (Runx2) and osterix (OSX) are important transcription factors in osteoblast differentiation and control the expression of bone-related genes such as OPN, BSP, and OCN (Okamura et al., 2013). BMSC-Exos up-regulates the expression of Runx2, OSX, OCN and other osteogenic genes (Yang et al., 2013; Tsao et al., 2017).

#### 3.1.1 Effects of BMSC-Exos on osteoblasts by protein

The expression of DNA-binding protein SATB2 (SATB2) in osteoblasts is beneficial for osteoblast differentiation (Gong et al., 2014). Wang et al. (Yang et al., 2019) treated osteoblasts with BMSC-Exos. It was found that the protein expression of SATB2 and cyclic AMP-dependent transcription factor 4 (ATF4) increased significantly. However, the expression of homeobox protein Hox-A2 (Hoxa2) was significantly decreased. The results showed that the changes in the expression of these proteins promoted osteoblast differentiation and bone regeneration. In a study on the effect of hypoxia-inducible factor 1α in BMSC-Exos (BMSC-Exos-HIF1α) on repairing critical bone defects in rats, it was found that BMSC-Exos-HIF1α stimulated osteogenic proliferation and differentiation of BMSCs (Ying et al., 2020). The combination of BMSC-Exos-HIF1α and beta-tricalcium phosphate (β-TCP) artificial bone can repair bone defects by promoting new bone regeneration and new blood vessel formation (Ying et al., 2020).

In summary, BMSC-Exos affects osteogenic differentiation and proliferation by targeting osteoblast delivery of carried proteins.

#### 3.1.2 Effects of BMSC-Exos on osteoblasts by non-coding RNA

MiRNA regulates about one third of the protein coding genes in human body, which has attracted extensive attention in terms of molecular regulation of gene expression (Zhang et al., 2015). The proportion of miRNA in exosomes is higher than that in metrocyte. Exosomes regulate osteoblast proliferation through miRNA, and affect mRNA expression. MiRNA-mediated dysregulation is an important pathological factor in osteoporosis and other bone related diseases (Wei et al., 2021).

In recent years, exosomes have been found to play an important role in regulating BMSCs osteogenic differentiation through miRNA. Xu et al. (2014) studied miRNA expression profiles in exosomes during BMSCs osteogenic differentiation and found that nine miRNAs significantly increased in BMSC-Exos, including let-7a, miR-218, miR-199b, miR-135b, miR-148a, miR-203, miR-299-5p, miR-302b and miR-219, etc. MiRNAs were found in exosomes during BMSC osteogenic differentiation. MiR-199b may regulate osteoblast differentiation through Runx2. Let-7b enhances osteogenesis and bone formation by regulating high mobility group protein A2, while inhibiting adipogenesis in human mesenchymal stem cells (hMSCs). MiR-218 promotes osteogenic differentiation of human adipose-derived mesenchymal stem cells through Wnt/β-catenin signaling pathway. MiR-135b regulates the mineralization of human stem cell osteogenic differentiation, which is up-regulated by impaired osteogenic differentiation of mesenchymal stem cells (MSCs) derived from multiple myeloma patients (Xu et al., 2014). Therefore, these results may indicate that exosomes miRNAs play a regulatory role in osteogenic differentiation through networking with cellular signaling pathways. Xu et al. (2014) also found that miR-221, miR-155, miR-885-5p, miR-181A and miR-320c were significantly decreased in the expression of BMSC-Exos. Down-regulation of miR-221 may lead to osteogenic differentiation of hMSCs. MiR-885-5p and miR-181a inhibited osteoblast differentiation Runx2 and TβR-I/Alk5, respectively. Therefore, down-regulation of miR-885-5p and miR-181a can promote the proliferation and activation of osteoblasts, thus becoming negative regulators of BMSCs osteogenic differentiation (Bhushan et al., 2013). In animal experiments, miR-196a enriched in BMSC-Exos promoted bone formation in Sprague-Dawley rats (SD rats) with skull defects (Qin et al., 2016). This study provides new directions for the research and treatment of exosomes secreted by BMSCs in the field of bone related diseases in the future.

Long non-coding RNA (lncRNA) is important in regulating various cellular physiological activities such as cell proliferation, differentiation, maturation and apoptosis. Yang et al. (2019) found that lncRNA Metastasis associated lung adenocarcinoma transcript 1 (MALAT1) in BMSC-Exos can be transferred to osteoblasts. Moreover, transfer-related lncRNA MALAT1 up-regulates the expression of SATB2 in osteoblasts by sponging miR-34c, thus regulating osteoblast differentiation.

**FIGURE 1:**
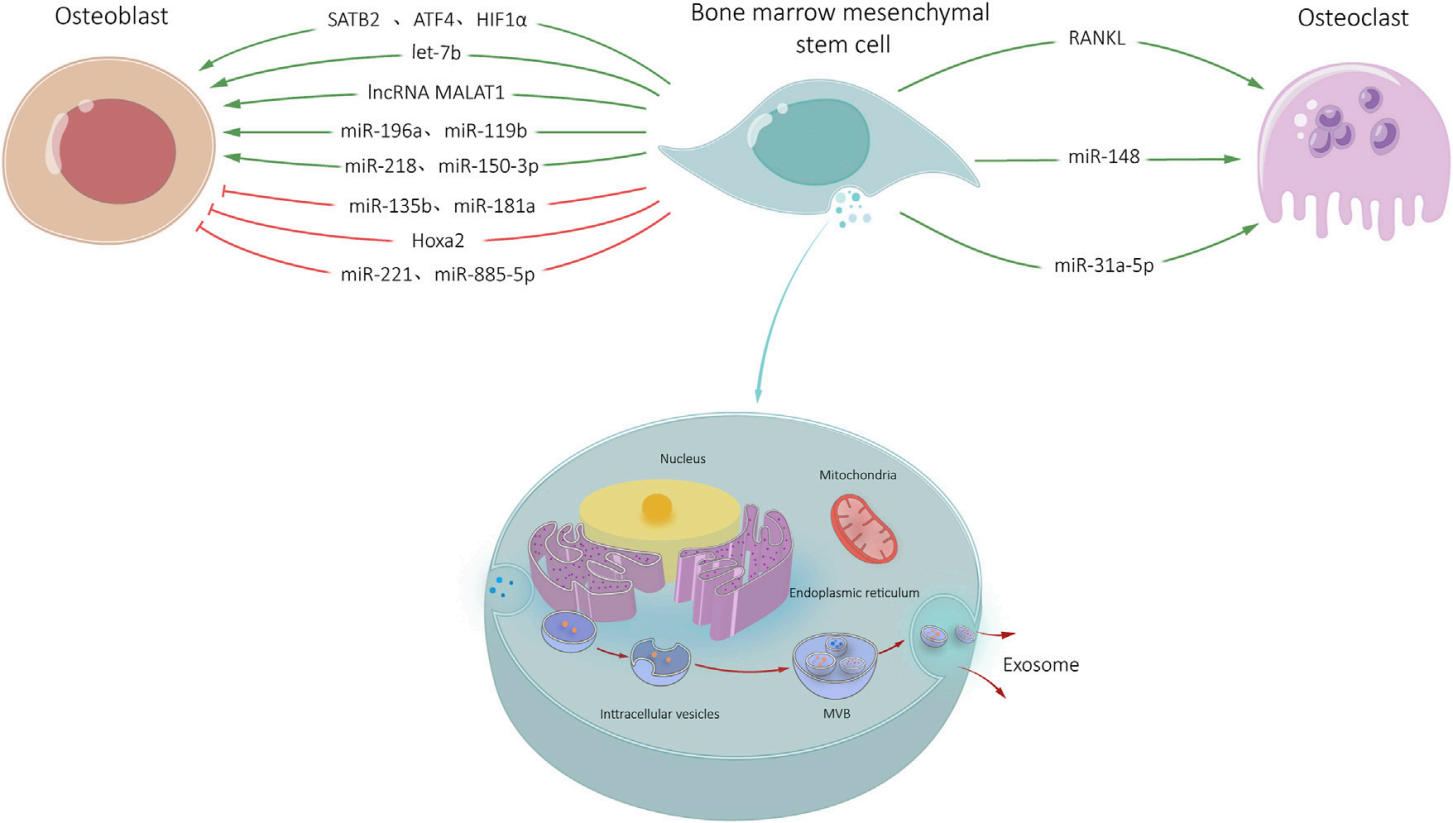
BMSC-Exos-mediated communication within the skeletal micro-environment. BMSC-Exos promoted osteoblast differentiation by SATB2, ATF4, HIF1*α*, let-7b, lncRNA MALAT1, and miRNAs (i.e., miR-196a, miR-119b, miR-218, miR-150-3p). BMSC-Exos inhibitied osteogenic differentiation through miR-135b, miR-181a, Hoax2, miR-221, and miR-885-5p. BMSC-Exos promoted osteoclast differentiation through RANKL, miR-148, and miR-31a-59.

Circular RNA (circRNA) was discovered in plant viruses in 1976 and has been considered as a byproduct of transcription without any other biological functions. However in recent years, circRNA has been found to widely exist in the cytoplasm of different organisms with high stability and play an important role in eukaryotic cells (Danan et al., 2012). In malignant tumors, circRNA binds to corresponding miRNA through miRNA response elements and inhibits gene expression, thus affecting the tumor development (Alimirzaie et al., 2019). However, there have been no reports on circRNA in BMSC-Exos for osteoblasts, suggesting a potential area for future research (Figure 1).

### 3.2 Effects of BMSC-Exos on osteoclasts

Osteoclasts are closely related to bone resorption and are mainly differentiated from macrophages and peripheral monocytes under the combined stimulation of multiple signaling factors. Exosomes are an important regulator of paracrine secretion of osteoclasts. RANKL plays a key role in osteoclast differentiation. RANK is abundantly expressed by exosomes in osteoclasts (Xu et al., 2018) and binds to RANKL to competitively inhibits the RANK pathway.

MiR-148a and miR-31a-5p in BMSC-Exos play an important role in osteoclast differentiation. MiR-148a promotes osteoclast differentiation by controlling transcription factor MAFB (MAFB) (Cheng et al., 2013). MiR-31a-5p regulates osteogenic differentiation of BMSCs and inhibits transforming protein RhoA (RhoA) expression by binding non-coding regions. MiR-31a-5p has been found to target regulatory coupling points between osteoclasts and osteoblasts (Xu et al., 2018). Inhibiting miR-31a-5p reduces bone resorption in osteoclasts, while enhancing bone structure (Xu et al., 2018). Therefore, inhibiting miR-31a-5p can alleviate bone metabolism imbalance in osteoporosis. However, no detailed reports have been reported on the regulation of lncRNA and circRNA in BMSC-Exos on osteoclasts. In a word, the results demonstrate the efficiency of BMSC-Exos in targeting osteoclasts to regulate bone resorption to promote bone regeneration, providing a novel approach for the treatment of osteoporosis (Figure 1).

## 4 Osteoblast-Exos-mediated communication within the skeletal micro-environment

BMSCs can differentiate and mineralize osteoblasts (MOB). BMSCs differentiate into bone progenitor cells, osteoblast precursors, and then form osteoblasts. Osteoblasts migrate to the absorbed site and secrete bone matrix. The bone matrix then mineralizes to form new bone. However, the role of MOBs in regulating MSCs remains unclear.

### 4.1 Effect of osteoblast-Exos on BMSCs

Exosome mRTNA translation into functional proteins control the fate of BMSCs. BMSCs co-cultured with MOB showed increased osteogenic ability *in vitro*, indicating the presence of soluble osteogenic factors released by MOB (Heino et al., 2004; Csaki et al., 2009; Birmingham et al., 2012; Tsai et al., 2012). In permineralized osteoblast cell line MC3T3-E1, exosomes promote the differentiation of BMSCs into osteoblasts (Cui et al., 2016). The activation of wnt/*β*-catenin signaling pathway promotes bone differentiation of mouse BMSCs. Exosomes isolated from osteoblasts contain key transcription factors involved in osteogenesis (Runx2 and OSX) (Narayanan et al., 2018). Extracellular matrix (ECM)-mediated BMSCs differentiation is enhanced by specific transcription factors and miRNA in osteoblast-Exos (Narayanan et al., 2018). SiRNA-mediated Runx2 knockdown further confirms the importance of exosomes RNA in lineage specific promoter activation (Mishima et al., 2021).

### 4.2 Effect of osteoblast-Exos on osteoclasts

Spinal tuberculosis is the main pathological feature of progressive bone destruction, and has the highest incidence rate in osteoarticular tuberculosis (Khanna and Sabharwal, 2019). Abnormal proliferation and activation of osteoclasts in spinal tuberculosis trigger pathological bone destruction. Osteoblasts and osteoclasts work together to promote the healthy bone growth.

The bone (reconstruction) model is characterized by alternating stages of osteoclast destruction and osteoblast formation (Ducy et al., 2000). Bone is constantly renewing itself through remodeling, a coordinated system in which osteoclasts absorb equal amounts of ECM and are deposited by osteoblasts (Wei and Ducy, 2010). Under normal circumstances, osteoblasts form new bone and osteoclasts absorb old bone. In the skeletal microenvironment, osteoblasts and osteoclasts interact with each other through cytokines, chemical transmitters, and cell contact (Tamma and Zallone, 2012; Yuan et al., 2018). Osteoblasts and osteoclasts maintain a dynamic balance to ensure the healthy bone growth (Chen et al., 2018). In pathological conditions, this dynamic balance is disrupted, affecting both bone structure and function (Tamma and Zallone, 2012). The interaction between osteoblasts and osteoclasts has been gradually recognized.

Exosomes is the medium of intercellular communication that has attracted recent attention from research. While osteoclast exosomes can transport miRNA and inhibit osteoblast function (Li et al., 2016; Sun et al., 2016; Yang et al., 2020), osteoblasts exosomes can also induce osteoclast formation (Deng et al., 2015). This exosome-mediated intercellular communication between osteoblasts and osteoclasts may represent a new mechanism for bone modeling and remodeling.

Bone homeostasis is largely maintained by the cellular communication between osteoclasts and osteoblasts. *Mycobacterium tuberculosis* lysate (MTL) stimulates mouse osteoblasts. Osteoblast-derived exosomes induced by MTL (MTL-OB-Exos) were isolated and extracted. Osteoclast precursor RAW264.7 cells were induced by MTL-OB-Exos. It was found that MTL-OB-Exos enhanced the osteoclast formation abilities of RAW264.7 (Yuan et al., 2018; Khanna and Sabharwal, 2019). Exosomes derived from osteoblasts contain RANKL protein, which can specifically bind to RANK on osteoclast precursor cells and participate in the RANKL-RANK-OPG regulatory axis to enhance osteoclast differentiation and function (Deng et al., 2015).

Exosomes-derived miRNAs have a role in dynamic bone homeostasis (Xie et al., 2017). It has been reported that mineralized osteoblasts release exosomes containing miR-503-3p. The molecular mechanism of miR-503-3p in osteoclasts remains unclear. It was found that the isolation of exosomes and miR-503-3p from osteoblast supernatant inhibited the differentiation of osteoclast progenitors (Wang Q. et al., 2021). Meanwhile, Heparinase gene (Hpse), the target gene of miR-503-3p, inhibited osteoclast differentiation by down-regulating Hpse expression. Osteoblast-derived exosomes inhibit osteoclast differentiation through miR-503-3p/Hpse axis (Wang Q. et al., 2021).

**FIGURE 2:**
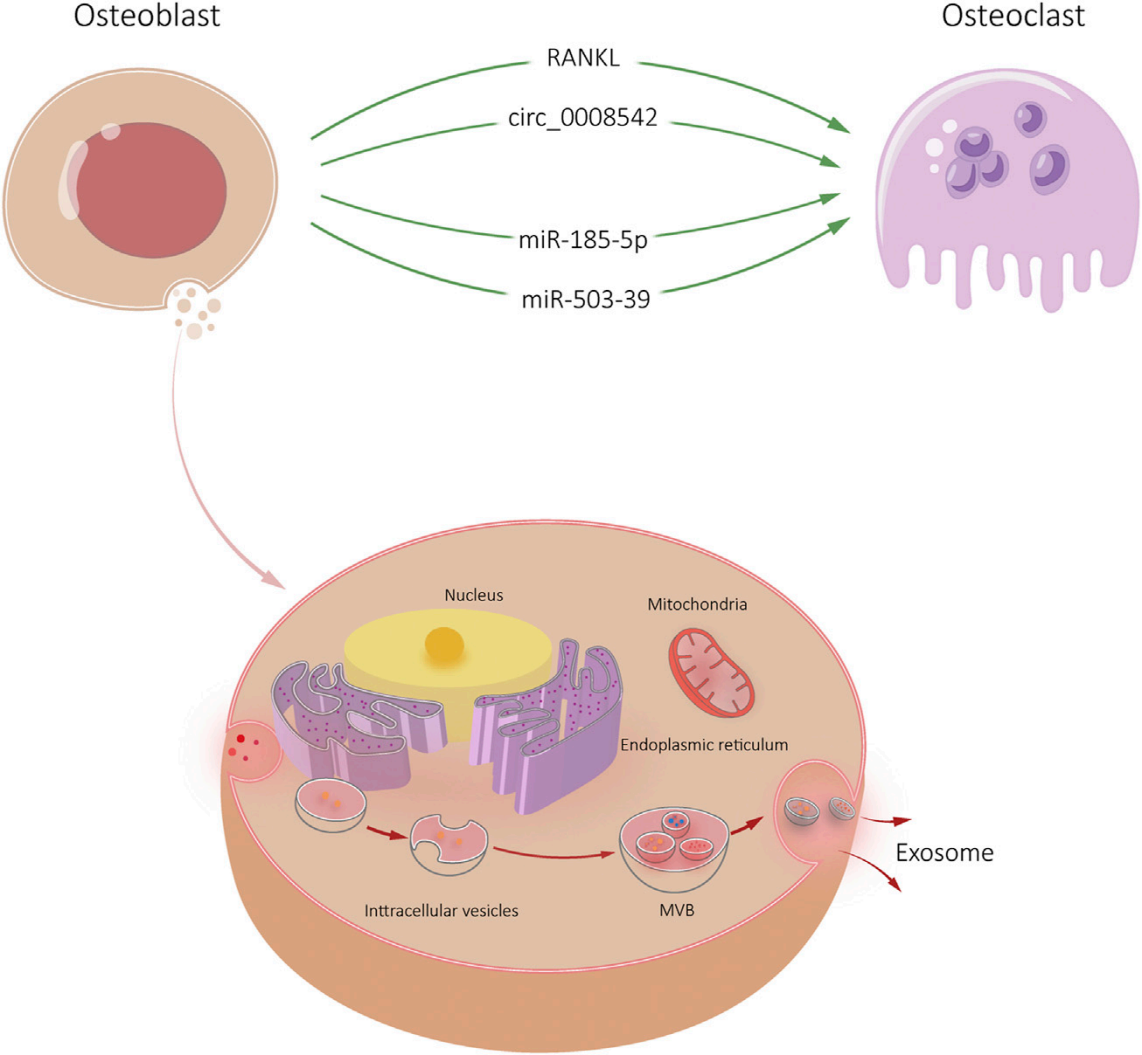
Osteoblast-Exos-mediated communication within the skeletal micro-environment. Osteoblast-Exos promoted osteoclast differentiation by RANKL, circ_0008542, miR-185-5p, and miR-503-39.

Current research on circRNA focuses on regulating cell transcription and translation, encoding proteins and molecular sponge activity of miRNA (Hansen et al., 2013; Memczak et al., 2013; Ashwal-Fluss et al., 2014). In skeletal system, circRNAs participate in the regulation of bone remodeling through the miRNA-mRNA axis in the form of molecular sponge (Qian et al., 2017; Li et al., 2018; Chen et al., 2019). The exosomes of MC3T3-E1 contain circ_0008542, which can increase tensile stimulation and promote osteoclast differentiation and bone resorption. Circ_0008542 upregulates Tnfrsf11a (RANK) gene expression by acting as miR-185-5p sponge. Specifically, circ_0008542 exerts a molecular sponge effect in osteoclasts through the miR-185-5p/RANK axis and gradually upregulates osteoclast differentiation (Wang W. et al., 2021). Meanwhile, circ_0008542 1916-1992bp fragment increased m6A methylation levels, inhibited RNA methylase METTL3, all of which induced osteoclasts differentiation and bone resorption. Injection of circ_0008542 + ALKBH5 into the tail vein of mice reversed the same effect *in vivo*. Site-directed mutagenesis showed that the 1956bp on circ_0008542 was the m6A functional site with the above biological functions (Wang W. et al., 2021). The RNA methylase METTL3 acts on the m6A functional site of 1956bp in circ_0008542, which promotes competitive binding of circ_0008542 to miR-185-5p. This binding results in increased expression of the target gene RANK and the initiation of osteoclast bone resorption. In contrast, the RNA demethylase ALKBH5 inhibits circ_0008542 binding to miR-185-5p, thereby correcting the bone resorption process (Figure 2).

## 5 Osteocyte-Exos-mediated communication within the skeletal micro-environment

Exosomes secreted by osteoblasts, osteocytes, osteoclasts, and other cells in the skeletal microenvironment influence bone formation and reabsorption. Also, exosomes affect bone tumor and lesion (Xie et al., 2017; Wang Q. et al., 2021; Hu et al., 2021). Osteocytes can regulate the functions of osteoblasts and osteoclasts by sensing systemic or local stimuli and secreting various cytokines and signaling molecules. Osteocytes derived exosomes contain many osteogenic factors, which can significantly enhance the targeted recruitment and osteogenesis of BMSCs.

It was found that miR-218 expression was significantly down-regulated in exosomes released by myostatin treated osteocytes (Qin et al., 2017). After endocytosis by osteocytes, Runx2 expression was down-regulated through the Wnt signaling pathway, thus inhibiting osteoblast differentiation. However, there was no significant change osteoclast activity of osteoclasts after exosome endocytosis (Qin et al., 2017). Sato et al. (2017) reported that the levels of 12 miRNAs, including miR-3473a, miR-6244, miR-5621-5p and miR-6239, in plasma exosomes of mice with reduced bone cells were significantly reduced. It is suggested that specific miRNA derived from osteocyte-Exos plays a biological role in bone remodeling. In addition, osteocyte-Exos carrying miR-124-3p can inhibit galectin-3 expression in osteoblasts under high glucose conditions, thereby reducing periodontal bone mass in diabetic periodontitis mice (Li et al., 2020). Whether this relevant mechanism is applicable in the treatment of diabetic OP still needs further experimental verification.

**FIGURE 3:**
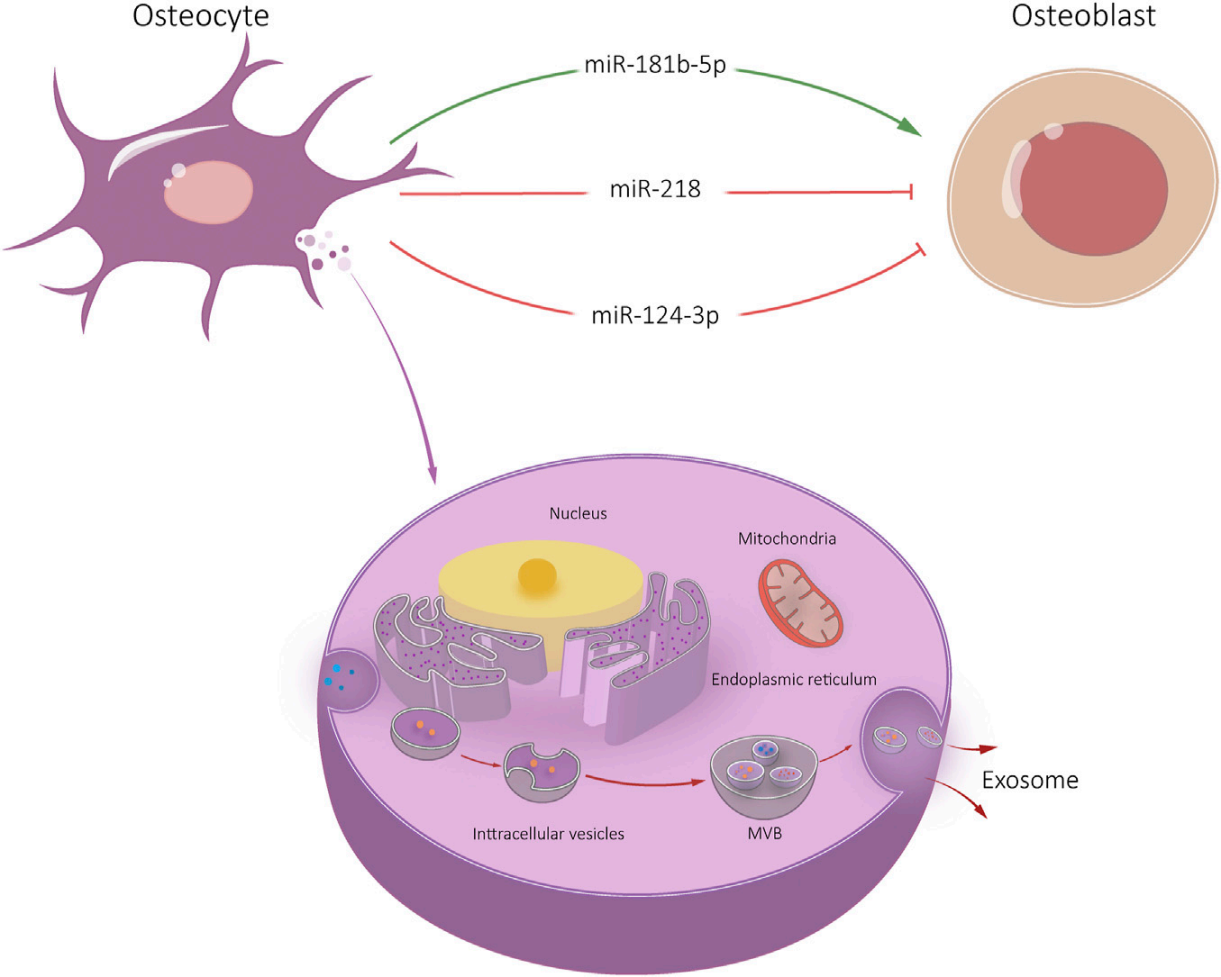
Osteocyte-Exos-mediated communication within the skeletal micro-environment. Osteocyte-Exos promoted osteoblast differentiation through miR-181b-5p, and inhibited osteoblast differentiation through miR-218 and miR-124-3p.

Osteocytes are mechanically sensitive cells that respond to external stimuli by regulating their secretion groups. Exosomes exposed to mechanical strain facilitate proliferation and osteogenic differentiation of human periodontal ligament stem cells (HPDLSC). High-throughput miRNA sequencing showed that miR-181b-5p was upregulated in exosomes exposed to mechanical strain. It may inhibit phosphatase and tensin homolog (PTEN). Meanwhile, protein kinase B (Akt) was enhanced (Lv et al., 2020). However, the research on osteocyte is still relatively new. Further research on osteocyte-Exos can provide a foundation for understanding bone remodeling and bone diseases (Figure 3).

## 6 Osteoclast-Exos-mediated communication within the skeletal micro-environment

Inflammatory bone disease is caused by bone loss caused by abnormally activated osteoclasts. Osteoclasts phenotypes and functions vary based on precursor cell origin, cytokine expression, and microenvironment-dependent factors. Inflammatory osteoclasts (iOCLs) lose their immunosuppressive effect relative to OCL under physiological conditions, which induces TNF-*α*-producing CD4^+^T cells in an antigen-dependent manner. This change ultimately leads to iOCL cascade amplification.

Osteoclasts-derived exosomes have been shown to modulate OCL-osteoblast communication (Yuan et al., 2018). iOCL-derived and OCL-derived exosomes promote osteoblast activity. We found that iOCL exosomes specifically target osteoblasts through ephrinA2/EphA2 (Ren et al., 2022). IOCL exosomes were rich in lncRNA from iOCL-derived exosome (lncRNA LIOCE), which promoted osteoblast activity after incorporation into osteoblasts. In addition, exosomes from lncRNA LIOCE stabilized osteogenic transcription factor OSX by reducing ubiquitination levels of OSX. Bone loss in a mouse model of inflammatory osteolysis was alleviated after injection of iOCL exosomes coated with lncRNA LIOCE. The role of lncRNA LIOCE-coated exosomes in promoting bone formation has been confirmed in rat bone repair models (Ren et al., 2022). IOCL-derived exosomes lncRNA LIOCE promote bone formation by upregulating OSX expression (Ren et al., 2022). Therefore, lncRNA LIOCE-coated exosomes may be an effective strategy for the treatment of osteoporosis and other bone metabolic diseases.

Osteoclasts secrete microRNA-rich exosomes through which miR-214 is transferred to osteoblasts to inhibit their function. MiR-214 and ephrinA2 levels are upregulated in serum exosomes of both patients with osteoporosis and transgenic mice models (Sun et al., 2016). These exosomes significantly inhibit osteoblast activity. Li et al. (2016) found that increased levels of osteoclast miR-214-3p and serum exosome miR-214-3p were associated with decreased bone formation in older women with fractures and ovariectomized mice. Osteoclast-specific miR-214-3p in knockout mice showed increased and bone formation decreased, suggesting that osteoclast exosomes affect osteoblast bone formation (Li et al., 2016).

Osteoclast targeting antagomir-214-3p therapy can rescue bone formation. Sun et al. (2016) found that exosomes of OCL recognized osteoblasts by ephrin A2/EphA2 and released miR-214/miR-214-3p into osteoblasts, inhibiting osteoblast differentiation. Yang et al. (2020) found that miR-23a-5p was highly expressed in the exosomes of RANKL induced RAW 264.7 cells. ALP staining showed that miR-23a-5p inhibits osteoblast activity.

**FIGURE 4:**
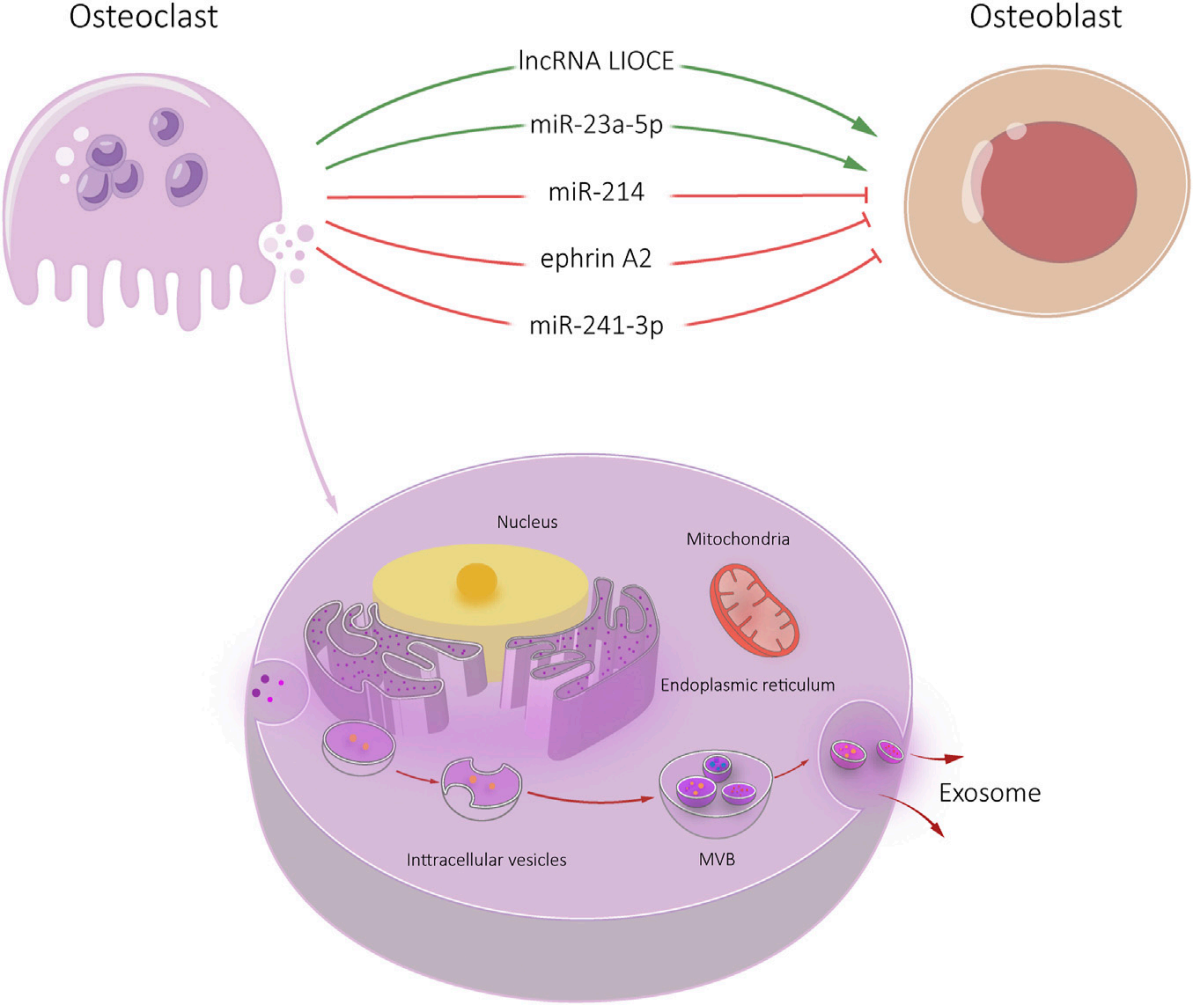
Osteoclast-Exos-mediated communication within the skeletal micro-environment. Osteoclast-Exos promoted osteoblast differentiation by lncRNA LIOCE and miR-23a-5p, and inhibited osteoblast activity by miR-214, ephrin A2 and miR-241-3p.

Osteoclast-derived exosomes containing miR-23a-5p can effectively inhibit osteogenic differentiation by inhibiting Runx2 and promoting transcriptional coactivator YAP (YAP)-mediated MT1DP. Runx2 is the target gene of miR-23a-5p, which interacts with YAP. Runx2 and YAP suppress putative metallothionein MT1DP (MT1DP) expression, which to promotes osteogenic differentiation by regulating Hepatocyte nuclear factor 3-*α* (FoxA1) and Runx2 (Yang et al., 2020) (Figure 4).

## 7 Summary

With the development of biomedicine, exosomes as drug carriers have attracted extensive attention of researchers (Yeo et al., 2013). Several exosome-based drug formulations are currently undergoing clinical trials, and some have recently been approved for clinical use. Exosomes have successfully encapsulated bioactive molecules such as curcumin, paclitaxel, neurotoxin-I, and dexamethasone, all of which improve biodistribution and controlled release (Chen et al., 2009; Hines and Kaplan, 2013). Exosomes can promote cell signaling *via* the endocrine or paracrine systems. Depending on the content within the exosomes (such as RNA, lipids, functional proteins and other active substances attached to them), exosomes can regulate the biological behavior of cells.

Transplantation of BMSC-Exos showed similar biological functions to BMSCs. BMSC-Exos can up-regulate the osteogenic gene expression and promote osteoblast proliferation and differentiation as well as bone regeneration. In BMSC-Exos, miRNA plays an important role in osteoclast differentiation. MiRNA can promote or inhibit osteoclast differentiation by binding specific non-coding regions. It is also a targeted regulatory point for coupling of osteoclasts and osteoblasts. Regulating miRNA in BMSC-Exos may be a potential method to attenuate the imbalance of bone metabolism in osteoporosis. Osteoblast-Exos can promote bone differentiation of BMSCs by activating Wnt/*β*-catenin signaling pathway. Specific transcription factors and miRNA in osteoblast-Exos enhance BMSCs differentiation. Osteoblast-Exos also serve as a medium for communication between osteoblasts and osteoclasts. Related lncRNA in osteoclast-Exos promote bone formation by up-regulating the expression of osteogenic genes. Related miRNAs in osteoclast-Exos are transferred into osteoblasts and affect osteoblast differentiation and bone formation. It is believed that the future studies on exosome of bone cells will play an important role in the occurrence and development of bone metabolic diseases and provide new ideas for fundamental research and clinical diagnosis of bone related diseases.
